# Integrative analysis of lithium treatment associated effects on brain structure and peripheral gene expression reveals novel molecular insights into mechanism of action

**DOI:** 10.1038/s41398-020-0784-z

**Published:** 2020-04-06

**Authors:** Amit Anand, Kunio Nakamura, Jeffrey M. Spielberg, Jungwon Cha, Harish Karne, Bo Hu

**Affiliations:** 1 Center for Behavioral Health, Cleveland Clinic, Cleveland, OH USA; 2 Department of Biomedical Engineering, Cleveland Clinic, Cleveland, OH USA; 3 Department of Psychiatry, University of Delaware, Cleveland, OH USA; 4 Center for Quantitative Health Sciences, Cleveland Clinic, Cleveland, OH USA

**Keywords:** Predictive markers, Molecular neuroscience

## Abstract

Lithium is a highly effective medication for bipolar disorder, but its mechanism of action remains unknown. In this study, brain MRI scans and blood samples for gene expression (total of 110 scans and 109 blood samples) were collected from 21 bipolar subjects before and after 2 and 8 weeks of lithium monotherapy and at the same time-points from untreated 16 healthy controls. We used linear mixed-effects models to identify brain structural features and genes with expression changed after lithium treatment, with correction for multiple testing, and correlated their concurrent changes to identify molecular pathways associated with lithium effects. There are significant increases in gray matter fraction, global cortical thickness, and the frontal and parietal cortices after 8 weeks of lithium treatment (corrected *p* < 0.05). Volume increases were also seen for putamen, hippocampus, thalamic nuclei, and thalamic substructures. Several genes showed significant expression changes, and 14 gene pathways were identified for the present integration analysis. Of these, nine pathways had significant correlations with structural changes (FDR < 0.05). Three neurotrophy-related pathways (GDNF family of ligands, NFAT immune-response, and p53-signaling pathway) correlated with structural changes in multiple regions. Mediation analysis showed that the sphingomyelin metabolism pathway is associated with HAM-D change (*p* < 0.01), and this effect is mediated via the volume of mediodorsal thalamus (*p* < 0.03). In summary, the integration of lithium effects on brain structural and peripheral gene expression changes revealed effects on several neurotrophic molecular pathways, which provides further insights into the mechanism of lithium action.

## Introduction

Lithium is a highly effective and specific medication for bipolar disorder (BD)^1–5^. Furthermore, it is a life-saving medication, as it has consistently been shown to decrease suicides as well as overall mortality in subjects who take it^6^. Though a number of molecular effects of lithium have been reported using in vitro experiments, their significance for clinical treatment and efficacy has been limited due to lack of suitable animal models for BD. This limitation has impeded methods to monitor its effects and the development of new medications with similar efficacy and specificity. Therefore, in vivo, clinical research using multi-modal data is needed to identify neurological and molecular biomarkers for lithium effects.

Basic science research on lithium has shown it to have a variety of acute effects on membrane function and signal transduction mechanisms^7^. In addition, it has been shown to affect several transcriptional regulators such as cAMP response element-binding protein (CREB), glycogen synthesis kinase 3 (GSK3), protein kinase C involved in signal transduction, and extracellular-regulated kinase (ERK)/mitogen-activated protein kinase (MAPK)^8–10^. Clinical effects of lithium on gene expression in animal models or in vitro in human cell lines^11,12^ have revealed changes in the expression of a number of genes involved in ion channel and receptor function, signal transduction (including the phosphatidylinositol system), neuroprotective mechanisms, energy metabolism, and thyroid function. However, the relationship of these changes in gene expression to clinical response to lithium in humans remains unclear.

In the past decade, gene expression in peripheral blood lymphocytes has been used as a proxy for central nervous system gene expression^13^. As brain biopsy is unrealistic in living humans, lymphocyte gene expression provides a convenient and accessible alternative to study molecular changes in health and disease, particularly if multiple samples are required^13,14^. Current evidence suggests that the vast majority of genes expressed in brain tissue are also found in blood, with tissue-specific expression occurring for only a small number of genes^15^. Recent studies have demonstrated that gene expression in peripheral blood lymphocytes is reasonably well correlated with expression in the central nervous system^16,17^. For example, nearly half of the candidate genes relevant to schizophrenia were expressed both in blood and prefrontal cortex, and expression levels of many biologically relevant classes of genes were not significantly different between blood and prefrontal cortex^16^. Changes in peripheral gene expression have now been used much more extensively, and recent reports show that these changes may help predict depression and suicidality^18,19^. Beech and colleagues conducted studies of gene expression change in peripheral lymphocytes in BD subjects on lithium treatment for one month^20^, as well as BD subjects prospectively treated with lithium for 6 months^21^. Responders had a greater upregulation of anti-apoptotic genes (BCL2) while pro-apoptotic genes were down-regulated. However, these studies were conducted while the subjects were simultaneously on other medications, and thus it is impossible to determine whether the effects observed were due to lithium.

Given the above limitations in the literature, we previously conducted a study of lithium monotherapy effect on peripheral lymphocyte gene expression and reported alterations in genes and pathways related to immune function, signal transduction, and regulatory molecules^22^. Thus, preliminary evidence of the molecular mechanisms of lithium is available. However, this research is limited in two important ways. First, the specific structural brain mechanisms by which these differences in gene expression impact pathology remain unknown. Second, gene expression was observed only peripherally, and thus there is no direct evidence that these changes had consequences in the brain. Given the difficulties discussed above regarding direct assessment of gene expression in the brain, it is necessary to simultaneously examine changes in peripheral gene expression and putative neural mechanisms to link these crucial levels of analysis.

In the present study, we used the same sample to investigate the relationship between changes in gene expression and changes in brain structure before and after lithium treatment^22^. Several studies have reported changes in brain structure after lithium therapy. Meta-analyses^23,24^, as well an analysis of data from an international consortium^25^, have provided further evidence in support of this finding. One major methodological issue in many studies is that they have used a cross-sectional design to compare structural volumetric differences between subjects on lithium for a variable period and subjects not on lithium. In many of these studies, subjects were also on other medications. However, a few studies which have used a longitudinal design to study lithium monotherapy effects, have also reported neurotrophic effects albeit variable results have been seen in a variety of regions using a small number of subjects^26–29^.

We hypothesized that integration of the structural changes with gene-expression pathways changes would provide more specific information regarding which molecular changes may be related to lithium mechanisms of action in BD. We examined global measures of brain structure (e.g., global cortical thickness), which should provide an overall assay of the impact of lithium on the brain. Given that changes may be more likely to occur in regions previously implicated in mood disorders, we also examined volumes in several specific brain areas. Thus, we hypothesized that lithium would be associated with increases in both global and regional indices. We also hypothesized that neural changes would correlate with gene expression changes in genetic pathways known to be implicated in lithium mechanisms of action. Furthermore, we hypothesized that the integration analysis of brain structure and gene expression would also reveal new molecular targets of the mechanism of action.

## Subjects and methods

### Participants

BD participants aged between 18 and 60 years were recruited via the Indiana University Hospital outpatient psychiatry clinic and community advertisement. All participants gave written informed consent, approved by the Institutional Review Board at the Indiana University School of Medicine.

All BD participants satisfied the DSM-IV-TR criteria for a current BD and current (hypo)manic or a depressed episode^30^. They were required to be medication-free for more than 2 weeks before study inclusion. Healthy subjects were required to have no personal or family history of psychiatric illness or alcohol or substance abuse dependence.

Further details of inclusion and exclusion criteria are presented in Supplementary Materials.

### Study design and lithium treatment

Baseline blood samples for gene expression analysis were collected after each participant signed the consent form. The participant also underwent a magnetic resonance imaging (MRI) scan in the same session.

Immediately after the baseline, BD participants started lithium treatment with 300 mg twice daily. Lithium levels were checked after about one week, and when necessary lithium dose was increased to achieve levels between 0.5 and 1.0 mEq/l. Blood sample collection and MRI scans were repeated for each participant after 2 and 8 weeks of treatment. Lithium levels were also checked near these follow-up visits.

The participants completed the 17-item Hamilton Depression Rating Scale (HAMD) and the Young Mania Rating Scale (YMRS)^31^ at baseline and weekly thereafter for the 8 weeks of lithium treatment. A psychiatrist assessed clinical improvement with the Clinical Global Impression of Severity (CGIS) and Improvement (CGII) Scale scores at week 2 and week 8 for overall bipolar illness^32^.

### RNA expression analysis

RNA expression analysis was conducted as described previously^22^, and details are given in the Supplementary materials.

### MRI acquisition and imaging analysis

*Details of structural MRI acquisition are presented in the* Supplementary materials.

T1w MPRAGE was preprocessed using iterative N3 intensity correction^33^ and standard space (International Consortium for Brain Mapping [ICBM]^34^) registration using a hierarchical approach^35^. Inter-session scans were co-registered using Medical Imaging NetCDF (MINC) toolkit V2 1.9.16^36^. Whole-brain fraction (WBF) was calculated as the ratio of brain parenchymal volume and outer brain contour volume, and the result was given in an arbitrary fractional unit. Gray matter fraction (GMF) and white matter fraction (WMF) were calculated using statistical parametric mapping (SPM) version 12 to segment MPRAGE image into gray matter, white matter, and cerebrospinal fluid^37^, and further combined with FSL (FMRIB Software Library) FIRST segmentation masks. The volumes of gray matter and white matter were divided by the intracranial volume, which was derived from the standard ICBM atlas to result in GMF and WMF in arbitrary unit.

Cortical thickness was measured using cortical longitudinal atrophy detection algorithm (CLADA)^38^, which was developed internally.

FSL’s FIRST (FSL version 5.0.9)^39^ was used to calculate the normalized volumes of accumbens, amygdala, caudate, hippocampus, pallidium, putamen, and thalamus on each hemisphere.

Percent change in the structures (WBF, GMF, WMF, and deep structures) was calculated using pairwise Jacobian integration technique^40^.

### Statistical analysis

The overall statistical data analysis consisted of two stages. Stage I identified significant volume changes from baseline to week 2 or 8 associated with the lithium treatment. Similarly, differential expression analysis was performed to identify genes with significant expression changes. Ingenuity pathway analysis (IPA) was then performed based on individual genes with significant expression changes (QIAGEN, Inc.). At week 2 or 8, for the genes or pathways and structural features with significant changes from baseline, Stage II correlated them pairwise to identify pairs associated with the lithium treatment together.

#### Lithium-induced structural change

Percent volume changes of each structural imaging feature at weeks 2 and 8 were analyzed using the linear mixed-effects model. Twenty-seven imaging features were tested, and features with Bonferroni corrected *p* < 0.05 were considered as having significant changes from baseline.

### Gene expression change

The gene expression data were first preprocessed, including transformation and normalization. Differential expression analysis was also performed using the linear mixed-effects model. The contrasts between baseline and week 2 or week 8 provide the estimated expression changes. The results were adjusted for multiple comparisons using FDR. The data from significant genes were then imported into IPA to identify canonical pathways. The gene expression of each IPA pathway was computed as the average of the normalized expression (i.e., *z*-score) of the genes involved in this pathway.

#### Integration of brain structural and gene expression changes

The integrative analysis had to be restricted to participants with both gene expression and MRI data measured at two time points (i.e., baseline and week 2 or 8). For each pair of gene and structure feature, the Pearson correlation coefficient was computed for the log 2-fold change of expression and the percent change of volume. The pairwise correlations were tested for statistical significance using FDR, and only the pairs with FDR < 0.05 were identified as having significant and correlated changes associated with lithium treatment. The same pairwise analysis was performed for pairs of IPA gene pathways and structure features. For each significant and correlated pair, mediation analysis was conducted with HAMD change as the outcome and the brain structure feature as the mediator using the *mediation* package in R^41^. All analyses were performed using R 3.6.0 (cran.r-project.org).

## Results

### Participant characteristics

Twenty-six BD participants had structural MRI scans at both baseline and week 8, and 25 of these had MRI scans at week 2. Among them, 21 (11 BPD and 10 BPM) participants had both MRI scan and gene expression performed. Demographic and illness characteristics of the population are presented in Table 1.

**Table 1:** Demographics and baseline illness characteristics for BD and healthy controls (HC) with both MRI and gene expression data.

Characteristic	BD (*N* = 21)	BPM (*N* = 10)	BPD (*N* = 11)	HC (*N* = 16)
Age (years)	33 (12)^a^	34 (14)	32 (11)	31 (9)
Female	11 (52.4%)	6 (60%)	5 (45%)	12 (75%)
Caucasian	21 (100%)	10 (100)	11 (100%)	14 (88%)
Bipolar I	10 (48%)	6 (60%)	4 (36%)	
HAMD score (17 item)	15 (8)	8 (3)	22 (4)	
YMRS score	9 (7)	16 (1)	2 (2)	
CGIS score	4 (0.2)	4 (0.3)	4 (0)	
Lithium levels—week 8	0.55 (0.2)	0.62 (0.3)	0.49 (0.1)	
Age at first episode (years)	13 (5)	12.5 (5)	13 (5)	
Medication free period (months)^b^	49 (83)	67 (116)	33 (36)	
Duration of current episode (weeks)	5 (5)	2 (3)	8 (5)	

Participants with both MRI and gene expression data.

^a^Statistics are mean (SD) for continuous variables and *N* (%) for categorical variables.

^b^Two participants were treatment naïve.

### Lithium effects on brain structure features

**Fig. 1:**
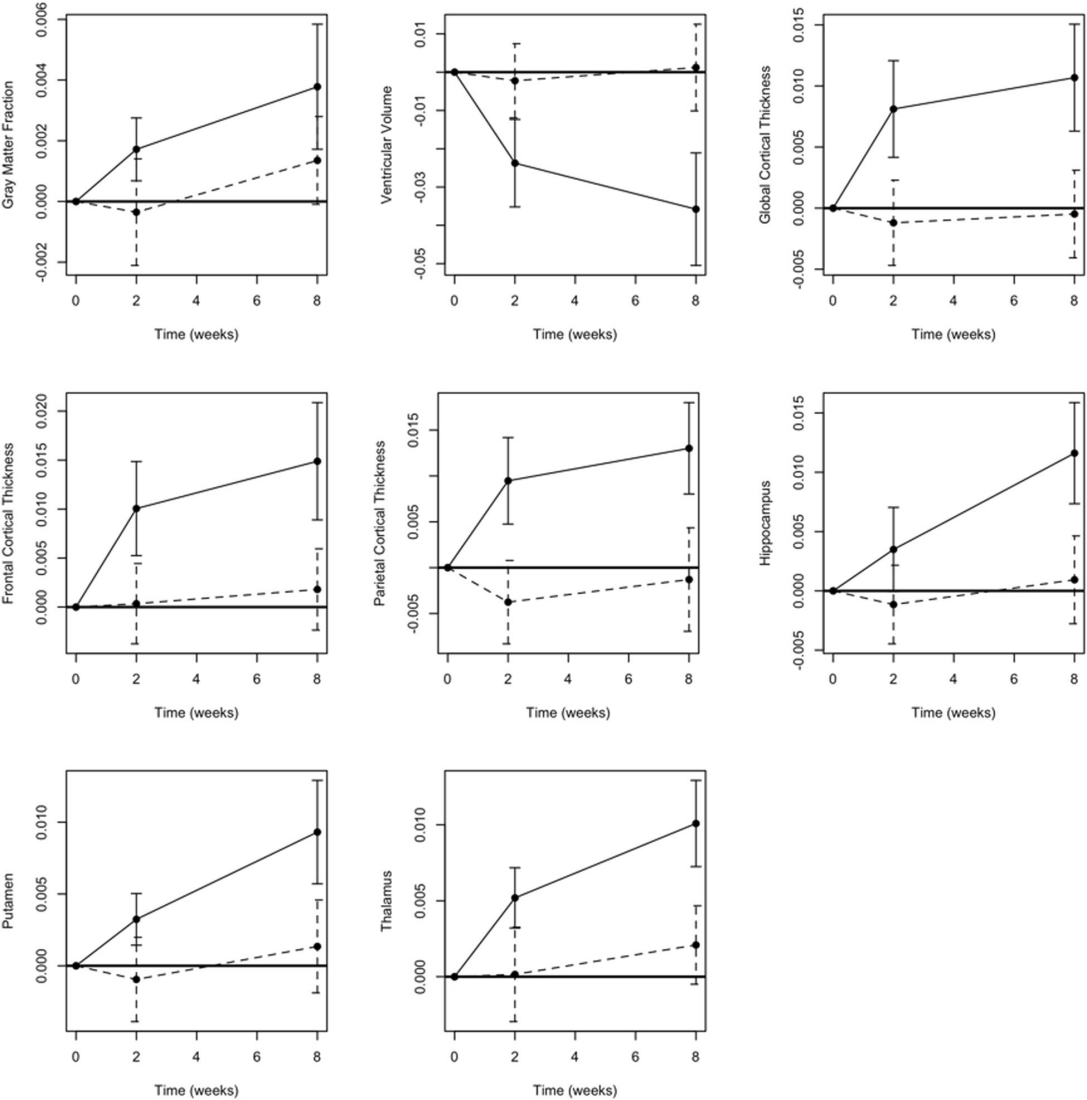
Trajectories of volumetric percent changes of brain structures by BD and HC subjects. Solid lines are for BD group and dashed lines are for HC group.

After 2 weeks of treatment, 8 structures had significant changes (Fig. 1 and Table 2), which include the ventricular volume and global, frontal, and parietal cortical thickness. These volume changes are −2.37%, 0.81%, 1%, and 0.94%, respectively, which were smaller than the changes at 8 weeks (please see below). The GMF increased by 0.17% (95% CI = [0.06%, 0.31%]), but this did not survive correction for multiple testing (*p* = 0.0837).

**Table 2:** Brain structure features with significant changes after 2 or 8 weeks of lithium treatment.

Feature	Baseline to Week 2		Baseline to Week 8	
	% Change [95% CI]	Corrected *p*-value^a^	% Change [95% CI]	Corrected *p*-value^a^
Gray matter fraction	0.17% [0.06%, 0.28%]	0.0914	0.38% [0.16%, 0.59%]	0.0395
Ventricular volume	−2.37% [−3.58%, −1.16%]	0.0118	−3.58% [−5.12%, −2.03%]	0.0020
Global cortical thickness	0.81% [0.39%, 1.22%]	0.0134	1.07% [0.61%, 1.53%]	0.0019
Frontal cortical thickness	1% [0.5%, 1.51%]	0.0110	1.49% [0.86%, 2.12%]	0.0015
Parietal cortical thickness	0.94% [0.44%, 1.44%]	0.0167	1.30% [0.78%, 1.82%]	0.0008
*Deep structure*				
Hippocampus	0.33% [−0.05%, 0.71%]	1.0000	1.16% [0.71%, 1.61%]	0.0005
Putamen	0.32% [0.13%, 0.51%]	0.0471	0.93% [0.55%, 1.31%]	0.0010
Thalamus	0.52% [0.31%, 0.73%]	0.0008	1.01% [0.71%, 1.31%]	7E−6
*Thalamus substructure*				
Anterior thalamus	0.65% [0.18%, 1.11%]	0.1806	1.20% [0.61%, 1.79%]	0.0095
Habenula thalamus	−1.74% [−2.9%, −0.57%]	0.1393	−2.19% [−3.45%, −0.92%]	0.0426
Lateral thalamus	0.93% [0.48%, 1.39%]	0.0077	1.79% [1.38%, 2.20%]	7E−8
Medial thalamus	0.69% [0.38%, 1%]	0.0028	1.16% [0.66%, 1.66%]	0.0021
Mediodorsal thalamus	0.74% [0.25%, 1.24%]	0.1172	1.18% [0.49%, 1.86%]	0.0454
Posterior thalamus	0.97% [0.16%, 1.79%]	0.5073	1.38% [0.60%, 2.16%]	0.0348

^a^Corrected for multiple testing using the Bonferroni approach.

After eight weeks of lithium treatment (Fig. 1 and Table 2), the GMF increased by 0.38% (uncorrected 95% CI = [0.16%, 0.59%], corrected *p* = 0.0377; Table 2). The WMF had no significant volume change (*p* = 0.95). The whole brain fraction increased by 0.23% (95% CI = [0.04%, 0.42%]), which was not significant after correction for multiple testing. The ventricular volume decreased significantly by 3.58% (95% CI = [2.03%, 5.12%], corrected *p* = 0.0018). Global mean cortical thicknesses increased by 1.07% (95% CI = [0.61%, 1.53%], corrected *p* = 0.0018). Frontal and parietal cortical thicknesses both had significant increases of 1.49% (95% CI = [0.86%, 2.12%], corrected *p* = 0.0014) and 1.30% (95% CI = [0.78%, 1.82%], corrected *p* = 0.0007), respectively.

Three deep structures had significant volume increases after 8 weeks of treatment. Specifically, putamen increased by 0.93% (95% CI = [0.55%, 1.31%], corrected *p* < 0.0001), hippocampus increased by 1.16% (95% CI = [0.71%, 1.61%], corrected *p* < 0.0001), and thalamus increased by 1.01% (95% CI = [0.71%, 1.31%], corrected *p* < 0.0001). For the thalamus, the volumes of six substructures changed significantly after the lithium treatment, including habenula, and anterior, lateral, medial, mediodorsal, and posterior thalamic nuclei. Habenula volume decreased by 2.19% (95% CI = [0.92%, 3.45%], corrected *p* = 0.041) while the volumes of other thalamus substructures all increased (Table 2).

Healthy subjects had no significant structural changes at either week 2 or 8 (Fig. 1). No significant differences were found between BPM and BPD patients.

### Lithium effects on gene expression

The differential gene expression analysis revealed results similar to the ones reported earlier^22^. Fifty-five out of 33,297 probes showed significant expression changes (FDR < 0.05) after 8 weeks of lithium treatment (Supplementary Fig. 1). Of these, 46 probes had significant expression increases, and the top genes included SLC31A2, IL5RA, P2RY14, FAR2, and TSPAN2; 9 probes had significant expression decreases, including top genes as CPT1A, PLB1, and FGGY. Twenty-three probes also had significant expression changes (FDR < 0.05) at 2 weeks. To identify gene *pathways* affected by lithium treatment, we applied a relaxed criterion by including genes with uncorrected *p* < 0.05, which provided an adequate number of genes for IPA. When comparing baseline to week 8, a total of 329 pathways were identified from IPA. Of these, we included 14 pathways which showed significant changes (uncorrected *p* < 0.01, see Supplementary Table 1) for the integration analysis. These pathways included several immune-related pathways: interferon signaling pathway, NFAT regulation pathway, GDNF family ligand–receptor interactions pathway, sphingomyelin metabolism pathway, and the pathogenesis pathway of multiple sclerosis. PIK3CG, PIK3R6, and ATM were the three most common genes found across these pathways.

For week 2, while 167 IPA pathways were identified, none of them had significant changes. Therefore, integration of gene pathways and structure features was not performed for week 2.

No gene had significant expression changes found for healthy subjects at either week 2 or 8. No significant differences were found between BPM and BPD patients.

### Integration of brain structural and gene expression changes

The integration analysis included 21 participants with both gene expression and MRI data. Supplementary Fig. 2 shows the correlations when integrating individual genes and structure features with significant changes at week 8. Positive correlations ranged from 0.43 to 0.65, whereas negative correlations ranged from −0.63 to −0.44. However, none of the correlations was significant (FDR < 0.05) after correction for multiple testing due to a large number of correlations. Also, no significant correlations were found when integrating individual genes and structure features at week 2.

**Fig. 2:**
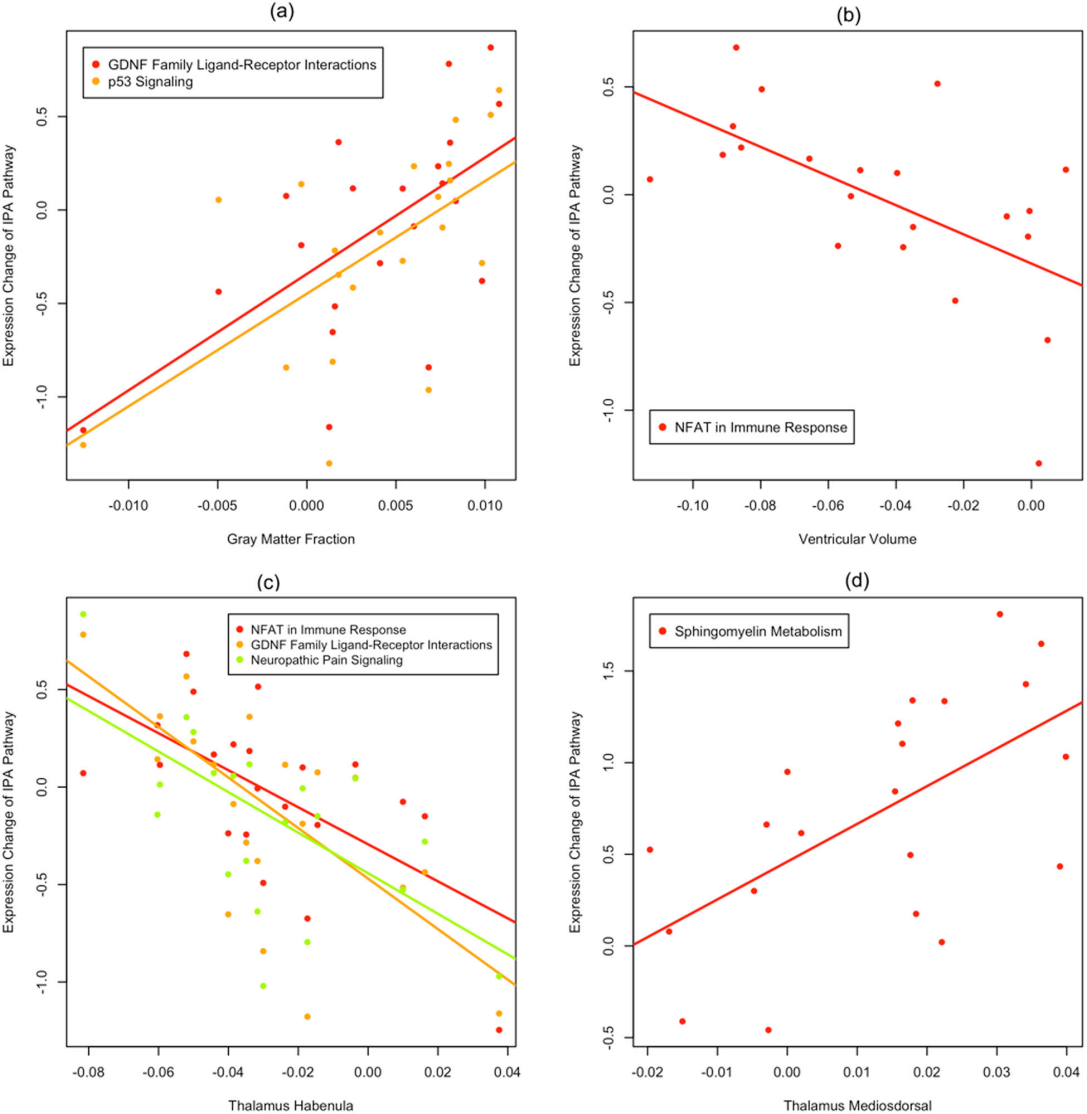
Scatter plots of expression changes of pathways significantly correlated with gray matter fraction, ventricular volume, and habenula and mediodorsal thalamus after 8 weeks of lithium treatment. *x*-axis represents percent change of the structure feature, and the *y*-axis represents pathway expression change (log 2-fold change from baseline).

Integrating gene *pathways* and structure features with significant changes at week 8 identified 23 pairs of pathway and structures with significantly correlated changes (FDR < 0.05). These 23 pairs involve 9 gene pathways and 7 structures (Table 3). Increase in GMF was positively correlated with increased expression of the GDNF family receptor pathway (*r* = 0.618, FDR = 0.033) and p53-signaling pathway (*r* = 0.602, FDR = 0.038) (Fig. 2a). The decrease in ventricular volume was negatively correlated with expression increases of the NFAT immune-response pathway (*r* = −0.592, FDR = 0.042; Fig. 2b). Figure 2c shows that habenula volume decrease was negatively correlated with increased expression of the GDNF pathway (*r* = −0.63), NFAT pathway (*r* = −0.614), and neuropathic pain signaling pathway (*r* = −0.583). Mediodorsal thalamus volume increase was positively correlated with increased expression of the sphingomyelin metabolism pathway (*r* = 0.598, FDR = 0.039).

**Table 3:** Gene pathways and structure features with significant correlated changes after 8 weeks.

IPA gene pathway	Structure feature	Correlation	FDR
Docosahexaenoic acid (DHA) signaling	Frontal cortical thickness	0.624	0.033
GDNF family ligand–receptor interactions	Gray matter fraction	0.618	0.033
	Global cortical thickness	0.675	0.019
	Frontal cortical thickness	0.703	0.015
	Habenula thalamus	−0.63	0.033
Neuropathic pain signaling in dorsal horn neurons	Global cortical thickness	0.649	0.028
	Frontal cortical thickness	0.665	0.022
	Habenula thalamus	−0.583	0.047
Non-small cell lung cancer signaling	Global cortical thickness	0.645	0.029
	Frontal cortical thickness	0.692	0.016
p53 signaling	Gray matter fraction	0.602	0.038
	Global cortical thickness	0.716	0.015
	Frontal cortical thickness	0.733	0.015
	Parietal cortical thickness	0.621	0.033
Role of NFAT in regulation of the immune response	Ventricular volume	−0.592	0.042
	Global cortical thickness	0.743	0.015
	Frontal cortical thickness	0.688	0.016
	Parietal cortical thickness	0.708	0.015
	Habenula thalamus	−0.614	0.033
Role of pattern recognition receptors in recognition of bacteria and viruses	Global cortical thickness	0.612	0.033
	Parietal cortical thickness	0.635	0.032
Sphingomyelin metabolism	Mediodorsal thalamus	0.598	0.039
UVA-induced MAPK signaling	Frontal cortical thickness	0.619	0.033

Global, frontal, and parietal mean cortical thicknesses were all positively correlated with the p53-signaling pathway and the NFAT immune-response pathway, and the correlations are mostly >0.7 (Supplementary Fig. 3). Global cortical thickness was also positively correlated with the GDNF family receptor pathway, the neuropathic pain signaling pathway, the non-small cell lung cancer signaling pathway and the pattern recognition receptors of bacteria and viruses.

### Mediated effects of gene pathways on HAMD changes

The mean HAMD score was 15 (SD = 8) at baseline, which decreased significantly (*p* = 0.002) to 11.6 after 8 weeks. Depressed participants had a mean decline of 10 (*p* < 0.001), while manic participants had a borderline significant increase of 3.8 (*p* = 0.051).

The mediation analysis showed that the expression change of the sphingomyelin metabolism pathway was significantly related with the percent change of the HAMD score, and the total effect is −0.514 (95% CI = [−0.943, −0.1], *p* = 0.01). A significant proportion of the effect was through the mediator of mediodorsal thalamus, which had an indirect effect of −0.321 (95% CI = [−0.660, −0.038], *p* = 0.022).

While most other pathways also had significant total effects on HAMD changes, none of them had both significant total and indirect effects (Supplementary Table 2). No significant results were found for the mediation analyses with YMRS scores.

## Discussion

The present study, the first to concurrently investigate brain structure and peripheral gene expression related with lithium treatment, revealed significant effects on both global and regional indices of brain structure. Crucially, these changes were found to correlate with peripheral expression pathways, and thus provide new information regarding the possible mechanism of action of lithium, in vivo, in the treatment of BD.

Lithium primarily led to increases in global GMF and mean cortical thickness, along with volume increases in frontal and parietal cortices. These findings are congruent with results from previous studies, which have shown an increase in global brain volume associated with lithium treatment^23,24,26–29^. In addition, increases in putamen, thalamus, and hippocampus volume were found. These areas are part of a putative mood regulation circuit, abnormalities of which may be present in BD^42–45^. The only area which showed a *decrease* in volume after lithium treatment was the habenula. The habenula receives input via the stria medullaris thalami and outputs to the many midbrain areas involved in releasing neurotransmitters, such as dopamine, norepinephrine, and serotonin^46^. It has been implicated in depressive behaviors^47^. The exact implication of lithium-induced decrease in habenular volume is not clear at this stage but could be related to inhibition of its function. We found that structural changes correlated with nine gene expression pathways, most of which have been implicated in neurotrophic function or cell modeling. Changes in three pathways were associated with volumetric changes in multiple brain structures (Table 3). The glial-cell-derived neurotrophic factor (GDNF) family receptor pathway expression correlated with increase in GMF and decrease in habenula volume. This pathway has been shown to promote the survival of many types of neurons, particularly dopaminergic neurons^48^, and has been investigated as a treatment of Parkinson’s disease^49^. Involvement of this protein-related receptor pathway in the mechanism of action of lithium is congruent with lithium’s purported neurotrophic properties.

The nuclear factor-activated T-cells (NFAT) immune response pathway increased expression correlated with increases in global, frontal, and parietal mean cortical thickness. NFAT has found to be important for neuroplasticity in both the developing and the adult brain^50^. NFAT has been reported to work with neurotrophic signaling to regulate axon outgrowth in several neuronal populations. The calcium-dependent calcineurin/NFAT-signaling pathway is also important in neuronal growth and axon guidance during vertebral development^50^.

The p53-signaling pathway was also found to correlate with increases in GMF and global, frontal, and parietal mean cortical thickness. Signaling in this pathway has been implicated in neuronal growth and has been investigated as a target to prevent neuronal degeneration^51^. p53 signaling was initially recognized to be involved in tumor suppression but currently is thought to be involved in all phases of cell development and growth.

Notably, we did not find significant changes in gene expression of dopamine-signaling pathways (though there was a trend for altered expression after 8 weeks of treatment *p* = 0.008 (uncorrected)), mitochondrial dysfunction/endoplasmic reticulum stress pathways, or AMPA receptor marker genes. In the future, larger studies are needed to specifically interrogate these biochemical mechanism pathways implicated in lithium mechanism of action.

Finally, mediation analyses revealed that the sphingomyelin metabolism pathway expression change was significantly correlated with percent change in HAM-D scores and that this effect was mediated via increases in mediodorsal thalamus volume. The mediodorsal thalamus is a nucleus within thalamus that is part of the putative mood circuit^42–45^. It receives inputs from the ventral striatum and outputs to the anterior cingulate cortex and the amygdala. The sphingomyelin pathway has been implicated in the pathophysiology of mood disorders. It has been suggested that sphingomyelin deposition in the hippocampus may be related to major depression and that antidepressant action may involve sphingomyelin/ceramide metabolism^52,53^. Thus, drugs targeting sphingomyelin metabolism may be useful in bipolar depression.

Limitations of the present study include the small number of subjects studied. The sample size also limits our ability to relate the findings to clinical response or remission. However, this is the first longitudinal study of its kind to collect both scan and gene expression data. Larger sample size studies need to be conducted in the future. A second limitation is that this study only provided information regarding short-term lithium effects. Longer duration effects (e.g., after 6–12 months) may provide more insight into long-term mood stabilization effects of lithium. This report focused only on structural volume and cortical thickness. Other studies need to be conducted to investigate molecular signatures of lithium effects on the functional and structural connectome implicated in the pathophysiology of BD. Finally, the purpose of the study was to investigate effects of lithium treatment in bipolar subjects. The study was not powered to find differences in structure between BP and healthy controls. We did not find any structure feature that was significantly different at a corrected significance value between BP patients and normal controls at baseline or week 8. We also compared BP patients at week 8 with normal controls at baseline, and the results also had no significant differences. Futures studies will need to be conducted with larger number of subjects to elucidate whether baseline structural differences between BP subjects and healthy controls exist and whether lithium leads to normalization of these differences if any.

## Conclusion

The present study provides indirect evidence regarding possible molecular pathways involved in lithium mechanism of action. These included GDNF, NFAT, p53, and sphingomyelin metabolism pathways, all of which are involved in neurogenesis and are consistent with known neurotrophic properties of lithium. In the future, these molecular targets could be explored for the development of therapeutic agents, which may have a similar effect as lithium in the treatment of BD.

## Supplementary information

Supplementary Material

Supplementary Figure 1

Supplementary Figure 2

Supplementary Figure 3
