# Supplementary material for: Integrative analysis of lithium treatment associated effects on brain structure and peripheral gene expression reveals novel molecular insights into mechanism of action
Source: Transl Psychiatry. 2020 Apr 6;10:103. doi: 10.1038/s41398-020-0784-z (PMC7136209; doi:10.1038/s41398-020-0784-z)
Supplement: Supplementary file 1 — Supplementary Material [file 41398_2020_784_MOESM1_ESM.pdf]

Supplementary Materials

Integrative Analysis of Lithium Effects on Brain Structure and Peripheral Gene Expression Reveals Novel Molecular Targets of Mechanism of Action

Amit Anand MD, Kunio Nakamura PhD, Jeffrey Spielberg PhD, Jungwon Cha PhD, Harish Karne MS, Bo Hu PhD

Summary

The material includes Subject Inclusion and Exclusion Criteria, Methods for RNA expression and MRI analyses, 3 supplementary figures, and 2 supplementary tables.

Inclusion and Exclusion Criteria

The exclusion criteria for BD participants included: lifetime diagnosis of schizophrenia or schizoaffective disorder; a current primary anxiety disorder; lithium in the past 6 months; use of psychotropic medications in the past 2 weeks; fluoxetine use over the past 4 weeks; acute suicidal or homicidal ideation or behavior; recent (<1 week) or current inpatient hospitalization; a diagnosis of substance dependence (except nicotine) within the past year; positive urinary toxicology screening at baseline; use of alcohol in the past 1 week; serious medical or neurological illness, and current pregnancy or breast-feeding.

Healthy subjects (18 – 60 y) additionally had no personal or family history of serious psychiatric or alcohol or substance abuse/dependence as determined by the Mini International Neuropsychiatric Interview or history of serious medical illness.

RNA Expression Analysis

Blood samples were collected in PaxGene tubes (2.5 ml), transported to the laboratory at room temperature and then stored at –20°C until processed using the Paxgene protocol (Qiagen).

Gene expression analysis was conducted in the Center for Medical Genomics at Indiana University as previously described.[20] Briefly, the samples were thawed and held at room temperature for 2 hours. The samples were centrifuged, and the supernatants removed.

The pellets were then treated with proteinase K at 65°C, then processed twice through PAXgene RNA spin columns, treated with DNase I, and processed again through PAXgene RNA spin columns. The RNA samples were stored at –80°C. Quantity and quality of the RNA was measured using a Nanodrop spectrophotometer and Agilent Bioanalyzer. RIN (RNA integrity number) values ranged from 5.7 to 9.6 (mean= 8.3, SD=0.67). One hundred-nanogram aliquots of each RNA were labeled and hybridized to an Affymetrix GeneChip® Human Gene 1.0 ST Array. The processing and labeling of samples were done in batches with all samples from a single individual processed together, and the individuals balanced for cases versus controls. All samples were hybridized individually. Scanned arrays were examined for defects and other quality issues.

MRI Acquisition

Scans were performed using a Siemens 3T Tim Trio. After a short scout imaging scan to survey head position and center the field of view (FOV), a high-resolution 3D magnetization prepared rapid gradient echo (MPRAGE) scan was performed and used for structural analyses. This high-resolution anatomical volume comprised of 160 sagittal slices and had 1.0×1.0×1.2 mm voxel dimensions, as optimized by the Alzheimer 's disease Neuroimaging Initiative protocol. The Scans were acquired with a repetition time (TR) 2300 ms, echo time (TE) 2.91ms, flip angel of 9° and FoV 240 x 256.

SUPPLEMENTARY FIGURE LEGENDS

Supplementary Figure 1. Volcano Plot of Differential Analysis for Gene Expression After 8 Weeks of Lithium Treatment in BD Subjects.

Supplementary Figure 2. Pairwise Correlations between Expression Changes of Significant Genes and Volume Changes of Significant Imaging Features After 8 Weeks of Treatment.

Supplementary Figure 3. Scatter Plots of Expression Changes of Pathways Significantly Correlated with (global, frontal and parietal) Cortical Thickness After 8 Weeks of Lithium Treatment/

Supplementary Table 1. Gene Pathways (IPA) With Significant Expression Changes After 8 Weeks of Lithium Treatment (p<0.01). These pathways were included in the integration analysis.

Pathway	Molecules	P-value
Interferon Signaling	IFIT3, IFIT1, IFITM3, OAS1, MX1, IFI6	2.14E-05
UVA-Induced MAPK Signaling	TIPARP, PIK3CG, PIK3R6, RPS6KA5, PLCL1, SMPD3, ATM	1.29E-03
Non-Small Cell Lung Cancer Signaling	STK4, FHIT, PIK3CG, PIK3R6, ITPR1, ATM	1.48E-03
Phenylethylamine Degradation I	AOC3, AOC2	1.51E-03
Role of Pattern Recognition Receptors in Recognition of Bacteria and Viruses	OAS1, IRF7, C3, PIK3CG, PIK3R6, C1QB, OAS3, ATM	1.70E-03
Role of NFAT in Regulation of the Immune Response	HLA-DOA, GNA15, PIK3CG, PIK3R6, FCER1G, RCAN3, ITPR1, FCGR1A, ATM	3.09E-03
Phagosome formation	RHOC, PIK3CG, PIK3R6, FCER1G, PLCL1, FCGR1A, ATM	3.63E-03
Histidine Degradation III	HAL, MTHFD2	6.76E-03
Sphingomyelin Metabolism	SGMS2, SMPD3	6.76E-03
GDNF Family Ligand-Receptor Interactions	DOK2, PIK3CG, PIK3R6, ITPR1, ATM	7.59E-03
Pathogenesis of Multiple Sclerosis	CXCL10, CXCL9	8.51E-03
p53 Signaling	TP53INP1, JMY, PIK3CG, PIK3R6, ATM, TP53I3	9.12E-03
Docosahexaenoic Acid (DHA) Signaling	ALOX15, PIK3CG, PIK3R6, ATM	9.55E-03
Neuropathic Pain Signaling In Dorsal Horn Neurons	CAMK1, PIK3CG, PIK3R6, ITPR1, PLCL1, ATM	1.00E-02

Supplementary Table 2. Total and Mediated Effects of Gene Pathways on Percent Change of HAMD (8 weeks)

	Mediator	Average Causal Mediated Effect[1]				Total Effect[1]			
Pathway	Structure Feature	Estimate	95% CI		P-value[2]	Estimate	95% CI		P-value
**Sphingomyelin Metabolism**	**Mediodorsal Thalamus**	**-0.321**	**-0.660**	**-0.038**	**0.022**	**-0.514**	**-0.943**	**-0.100**	**0.01**
Docosahexaenoic Acid (DHA) Signaling	Frontal Cortical Thickness	-0.329	-0.876	0.048	0.092	-0.397	-0.913	0.135	0.146
GDNF Family Ligand-Receptor Interactions	Gray Matter Fraction	-0.345	-1.117	0.230	0.26	-0.456	-0.890	0.000	0.052
	Global Cortical Thickness	-0.228	-0.689	0.097	0.21	-0.468	-0.954	-0.014	0.046
	Frontal Cortical Thickness	-0.240	-0.704	0.113	0.228	-0.462	-0.874	-0.071	0.014
	Habenula Thalamus	-0.148	-0.543	0.143	0.35	-0.480	-0.976	-0.037	0.032
Neuropathic Pain Signaling In Dorsal Horn Neurons	Global Cortical Thickness	-0.185	-0.678	0.146	0.308	-0.667	-1.193	-0.274	0.002
	Frontal Cortical Thickness	-0.167	-0.596	0.128	0.282	-0.666	-1.088	-0.262	0.002
	Habenula Thalamus	-0.114	-0.579	0.239	0.49	-0.668	-1.151	-0.187	0.008
Non-Small Cell Lung Cancer Signaling	Global Cortical Thickness	-0.172	-0.553	0.174	0.31	-0.611	-1.037	-0.211	0.002
	Frontal Cortical Thickness	-0.172	-0.576	0.166	0.326	-0.614	-0.997	-0.252	0.002
p53 Signaling	Gray Matter Fraction	-0.237	-0.720	0.082	0.164	-0.482	-0.884	-0.077	0.016
	Global Cortical Thickness	-0.230	-0.724	0.207	0.286	-0.465	-0.874	-0.064	0.026
	Frontal Cortical Thickness	-0.213	-0.712	0.242	0.372	-0.477	-0.866	-0.073	0.032
	Parietal Cortical Thickness	-0.183	-0.608	0.176	0.312	-0.482	-0.975	0.019	0.06
Role of NFAT in Regulation of the Immune Response	Ventricular Volume	-0.498	-1.152	-0.031	**0.028**	-0.577	-1.316	0.177	0.122
	Global Cortical Thickness	-0.350	-1.154	0.336	0.31	-0.597	-1.340	0.174	0.124
	Frontal Cortical Thickness	-0.312	-1.126	0.396	0.342	-0.583	-1.319	0.133	0.13
	Parietal Cortical Thickness	-0.285	-0.889	0.244	0.298	-0.589	-1.399	0.253	0.146
	Habenula Thalamus	-0.210	-0.831	0.380	0.46	-0.552	-1.481	0.341	0.214
Role of Pattern Recognition Receptors in Recognition of Bacteria and Viruses	Global Cortical Thickness	-0.438	-1.184	0.110	0.096	-0.429	-1.111	0.271	0.238
	Parietal Cortical Thickness	-0.421	-1.089	0.123	0.142	-0.401	-1.130	0.364	0.306
UVA-Induced MAPK Signaling	Frontal Cortical Thickness	-0.159	-0.593	0.197	0.38	-0.602	-1.100	-0.152	0.016

[1]Average Causal Mediated Effect (ACME)=pathway effect on HAMD change mediated by the structure feature; total effect=direct effect + ACME[42]

[2]p<0.05 for ACME indicates a significant mediation effect (i.e., structure feature mediates the gene pathway)

Supplementary Figure 1. Volcano Plot of Differential Analysis for Gene Expression After 8 Weeks of Lithium Treatment in BD Subjects

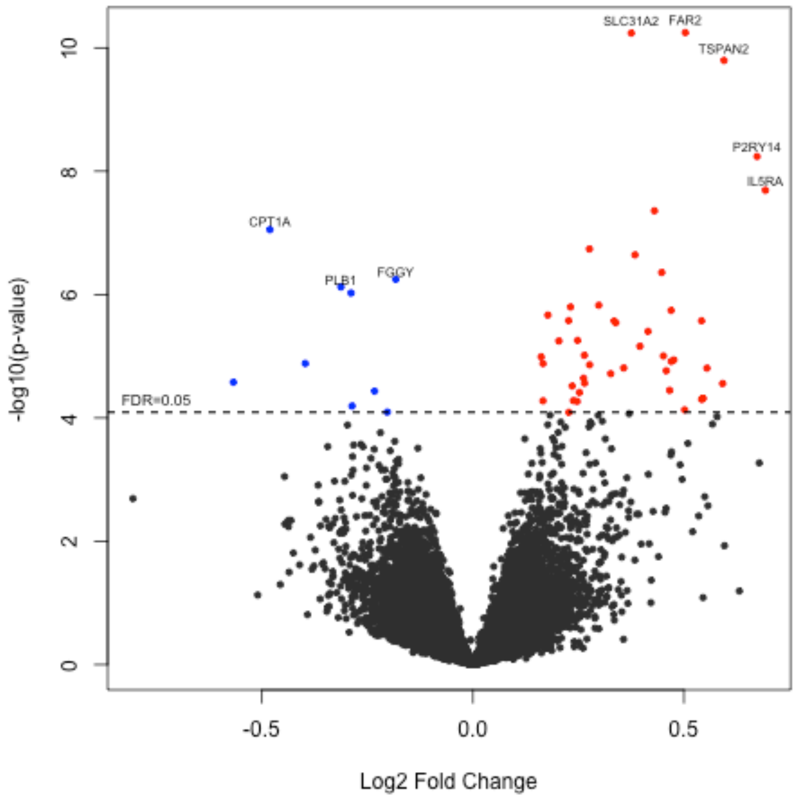

Supplementary Figure 2. Pairwise Correlations between Expression Changes of Significant Genes and Volume Changes of Significant Imaging Features After 8 Weeks of Treatment

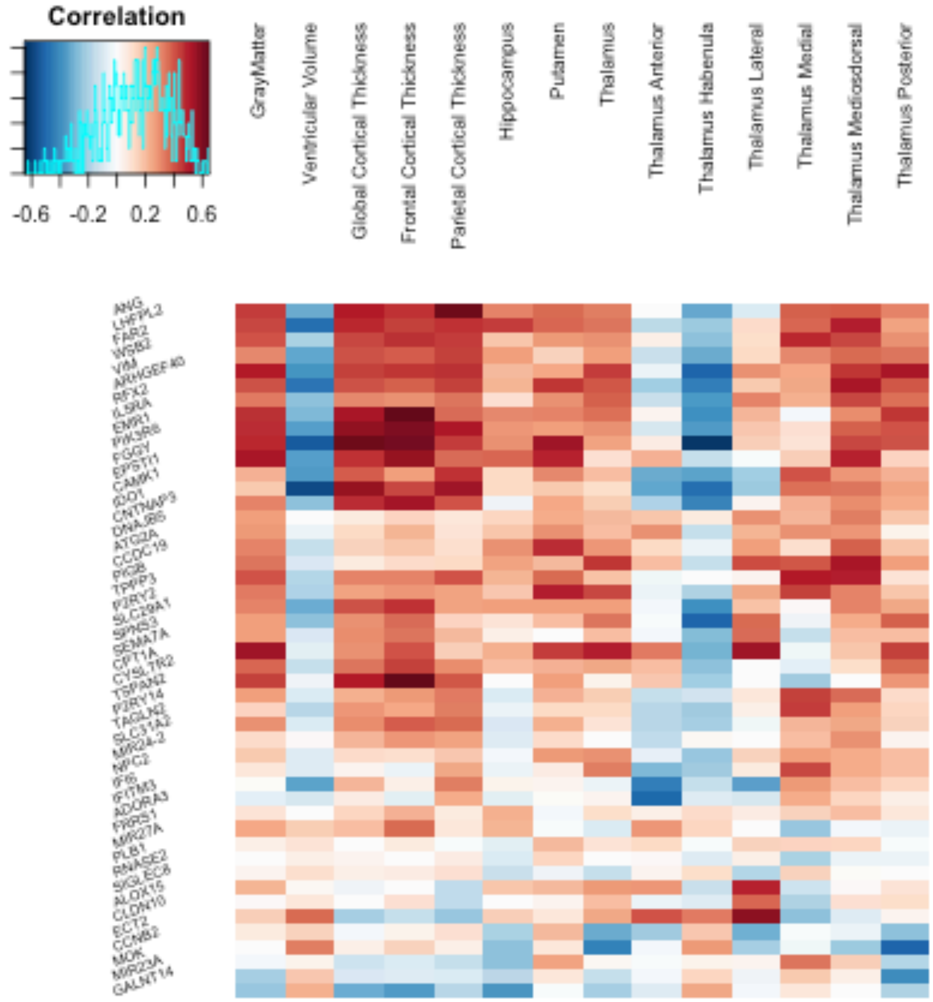

Supplementary Figure 3. Scatter Plots of Expression Changes of Pathways Significantly Correlated with (global, frontal and parietal) Cortical Thickness After 8 Weeks of Lithium Treatment

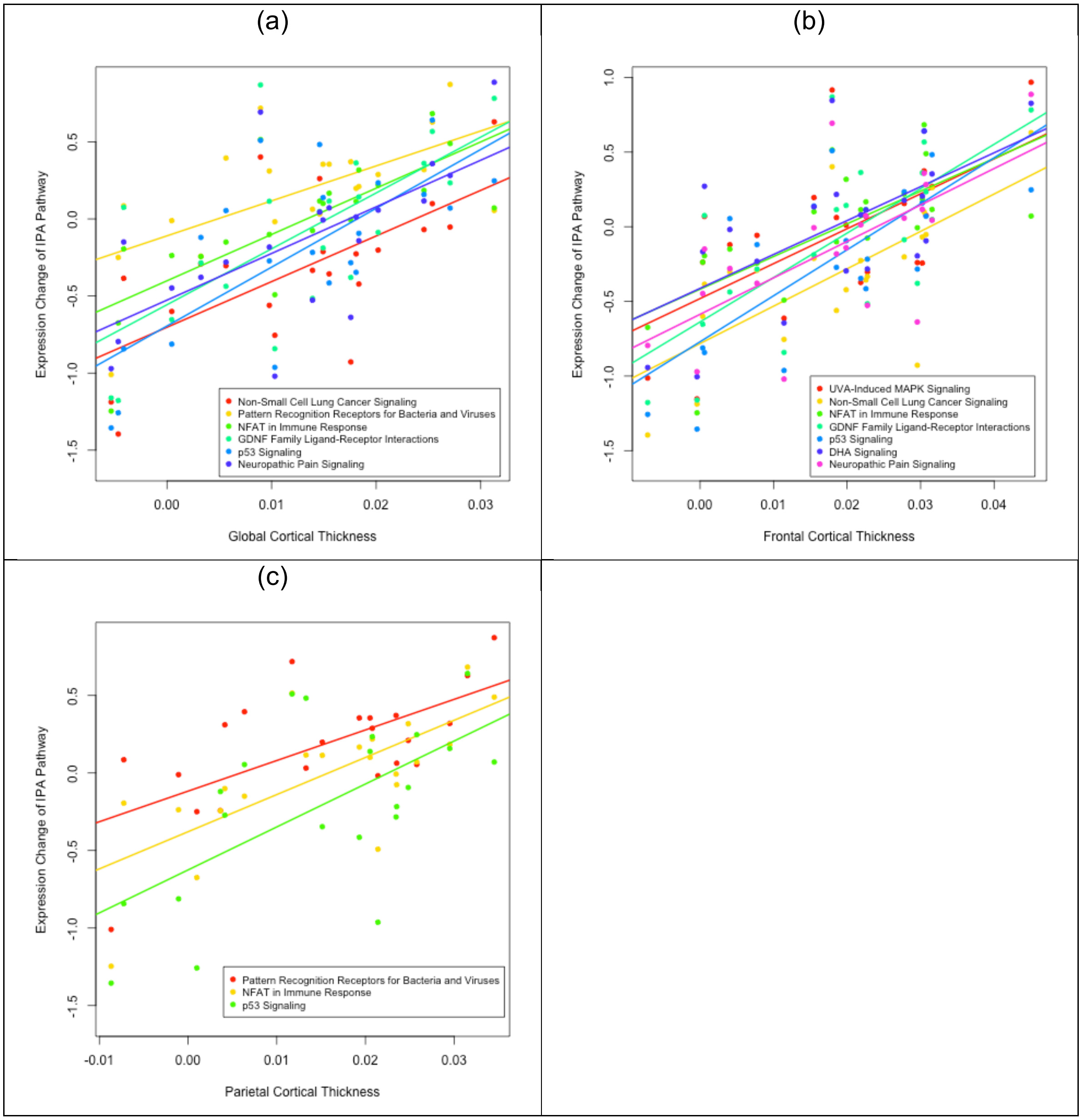
(a)
Expression Change of IPA Pathway
Global Cortical Thickness
Non-Small Cell Lung Cancer Signaling
Pattern Recognition Receptors for Bacteria and Viruses
NFAT in Immune Response
GDNF Family Ligand-Receptor Interactions
p53 Signaling
Neuropathic Pain Signaling
(b)
Expression Change of IPA Pathway
Frontal Cortical Thickness
UVA-Induced MAPK Signaling
Non-Small Cell Lung Cancer Signaling
NFAT in Immune Response
GDNF Family Ligand-Receptor Interactions
p53 Signaling
DHA Signaling
Neuropathic Pain Signaling
(c)
Expression Change of IPA Pathway
Parietal Cortical Thickness
Pattern Recognition Receptors for Bacteria and Viruses
NFAT in Immune Response
p53 Signaling
