# Supplementary figures and images for: Integrative analysis of lithium treatment associated effects on brain structure and peripheral gene expression reveals novel molecular insights into mechanism of action
Source: Transl Psychiatry. 2020 Apr 6;10:103. doi: 10.1038/s41398-020-0784-z (PMC7136209; doi:10.1038/s41398-020-0784-z)

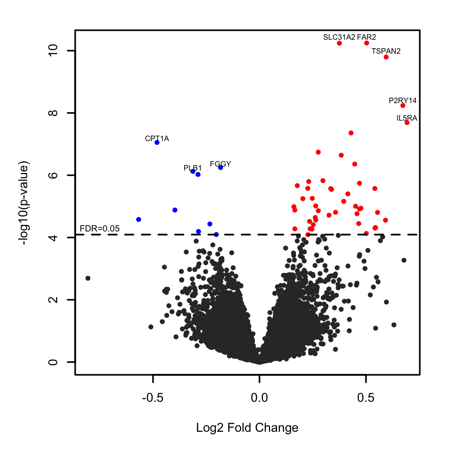

Supplement: Supplementary file 2 — Supplementary Figure 1 [file 41398_2020_784_MOESM2_ESM.tif]

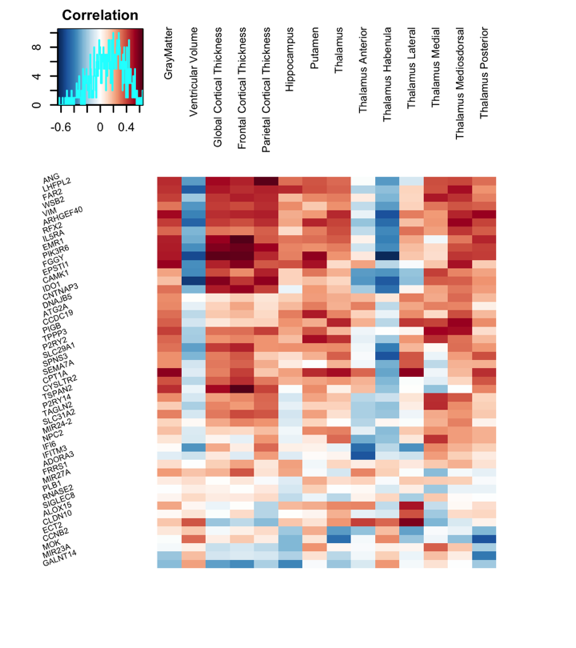

Supplement: Supplementary file 3 — Supplementary Figure 2 [file 41398_2020_784_MOESM3_ESM.tif]

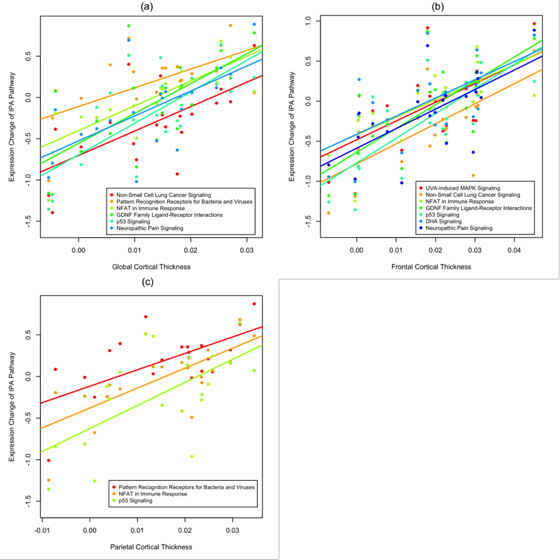

Supplement: Supplementary file 4 — Supplementary Figure 3 [file 41398_2020_784_MOESM4_ESM.tif]
